# Multiscale Modelling and Analysis of Collective Decision Making in Swarm Robotics

**DOI:** 10.1371/journal.pone.0111542

**Published:** 2014-11-04

**Authors:** Matthias Vigelius, Bernd Meyer, Geoffrey Pascoe

**Affiliations:** FIT Centre for Research in Intelligent Systems, Monash University, Melbourne, Australia; Arizona State University, United States of America

## Abstract

We present a unified approach to describing certain types of collective decision making in swarm robotics that bridges from a microscopic individual-based description to aggregate properties. Our approach encompasses robot swarm experiments, microscopic and probabilistic macroscopic-discrete simulations as well as an analytic mathematical model. Following up on previous work, we identify the symmetry parameter, a measure of the progress of the swarm towards a decision, as a fundamental integrated swarm property and formulate its time evolution as a continuous-time Markov process. Contrary to previous work, which justified this approach only empirically and *a posteriori*, we justify it from first principles and derive hard limits on the parameter regime in which it is applicable.

## Introduction

Swarm robotics refers to the concept of using a number of autonomous and often very simple robots to collaboratively accomplish a task. For some scenarios, this can provide an attractive alternative to deploying a single, complex robot. Typically, three main advantages of a swarm-based approach are cited, which can also be viewed as design goals [2]–[4]. (i) *Robustness*: swarm performance does not critically depend on individuals and degrades gracefully when individuals malfunction. (ii) *Scalability*: swarm behaviour can scale well for a wide range of problem sizes (and swarm sizes). (iii) *Flexibility*: swarms are assumed to adapt their behaviour flexibly to changing environmental conditions.

When designing swarm-control mechanisms, researchers and engineers are faced with the challenge to develop a set of rules at the individual (microscopic) level such that a desired behaviour at the group (macroscopic) level is achieved [4]. This is a very difficult task since there is no general systematic way to devise individual behaviours that reliably achieve a desired group behaviour. Thus design choices can usually only be tested in experiments or simulations. Performing swarm experiments is expensive and requires considerable effort and time comitment [3], [4]. Simulations, on the other hand, are efficient and fast but cannot achieve the same degree of realistic behaviour as physical experiments. Any approach that allows us to derive predictions of a swarm's behaviour analytically would thus be of significant advantage. In an attempt to address this, the present paper presents an approach that brings together physical experiment, simulation and analytic predictions.

Generally, swarm simulations are categorized depending on their level of abstraction [3]. (i) Microscopic simulations model the behaviour of individual robots and the interaction between robots. (ii) Macroscopic models describe the number of robots in the different behavioural states [5], [6]. It is useful to distinguish between macroscopic-discrete and macroscopic-continuous methods [7]. Discrete approaches model the count of robots in each one of a finite set of states, while continuous approaches model the (real-valued) fraction of the whole population in each of the states. Macro-discrete models are amenable to a master equation approach and thus typically treated stochastically [6], [8], [9], while macro-continuous approaches typically result from an averaging procedure [8] and are hence deterministic. A further possibility is to have an infinite number of states or continuous state variables. This can be treated deterministically or stochastically [1].

Ideally, a unified approach to modelling a robot swarm will derive parameters for a microscopic description from experiments, derive macroscopic equations from the microscopic model and perform microscopic simulations to validate the macroscopic description [4]. Several factors seem to make such an approach challenging. First, microscopic simulations, while more accessible than physical experiments, generally require substantial computational resources if they involve a large number of robots. Second, physical experiments are expensive, time-consuming and can usually only be conducted under sanitized laboratory conditions [4]. Third, deriving macroscopic descriptions from probabilistic microscopic ones is usually hard, in particular if spatial aspects need to be taken into account [8], [9].

The present paper explores the feasibility of such a unified approach using the example of a typical collective-decision making problem [1]. We show that, despite the above challenges, such an approach is feasible provided the process under investigation meets specific requirements. We derive our modelling method from first principles based on chemical kinetics. This enables us to analyze the requirements for its applicability and its limitations systematically and in detail. It also allows us to have confidence in the approach beyond a purely empirical justification if these requirements are met.

This article builds upon Hamann *et al.* [1]. It extends this work in several regards. Firstly, we present a consistent multi-scale approach that spans microscopic, macroscopic-discrete and macroscopic descriptions. Secondly, we make a first attempt at deriving the aggregate continuous-time Markov model from first principles. This was justified only *a posterori* in Hamann *et al.* [1]. We do so by transforming the microscopic equations for the individual agent motion into an aggregate macroscopic-discrete reaction system. This transformation is firmly grounded on established techniques from chemical kinetics. This approach vastly improves the confidence we may have in a continuous-time Markov description provided the system under consideration is within well-defined limits. Thirdly, using a mathematical toy model, we show a way to analytically derive the characteristic parameters of the stochastic differential equation for the aggregate model from the macroscopic-discrete description.

Several other authors in recent years have applied methods from chemical reaction networks to analyze the dynamics of robot swarms [10]–[15]. Like these earlier works our approach is rooted in the theory of reaction kinetics, but our main concerns differ from these works subtly but importantly. Firstly, we are interested in quantifying transient behaviour as much as steady-state behaviour, for example the time to reach a particular state. Previous work has often only been concerned with steady state behaviour [10], [13]. Secondly, we are specifically interested in how group behaviour emerges from local interactions and communication between swarm members. Previous work has often explicitly excluded local communication and interaction from the methodology [11], [12], [15]. Thirdly, we are interested in systematically abstracting, based on first principles, a spatially extended scenario into a non-spatial macroscopic model without excluding spatial inhomogenities that may emerge from local interactions. Previous work has not explicitly made this connection. [10] explicitly incorporate spatial inhomogenities but restrict these to the ones known a-priori, such as aggregation at a set of predefined boundaries.

The paper is structured as follows. We start by introducing the density estimation task and our implementation on a microscopic level using the multi-agent framework flame (http://www.flame.ac.uk/) in Section “Microscopic approach''. Here we also reproduce some of the results from [1]. In Section “Kilobot experiments'' we present results from a physical implementation of the density classification task using a swarm of Kilobots [16]. We then ask the question if the microscopic (agent-based) description can be translated into a spatially-homogeneous macroscopic-discrete formalism (Section “The macroscopic-discrete approach''). This section contains our derivation of the macroscopic-discrete Master equation and numerical validations using the software package inchman [17], [18]. Finally, we analytically solve the macroscopic-discrete Master equation for a simplified model and obtain expressions for the coefficients of the associated Fokker-Planck equation (Section “Constructing a time coarse-grained Markov process for the symmetry parameter''). In this way we can justify the aggregate continous-time Markov model and close the loop.

## Microscopic Approach: Virtual Swarm

### 1 Simulation setup

Density classification is a well-known example for the concept of embodied swarm computation, where a consensus emerges from local interactions [19]. The task consists of determining the majority color of a set of 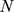 randomly-initialized (red or green), spatially distributed agents. This can be achieved by allowing the individuals to roam freely (with an initially randomly-distributed velocity 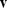) while constantly monitoring the position of nearby inviduals. If another agent enters the immediate proximity of the individual, where proximity is defined by the *avoidance radius* 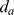, both agents turn around and remember the color of their collision partner. Once any agent performed 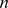 collisions, where the decision threshold 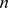 is a free parameter, it changes its color to the dominant color it encountered during collisions. This algorithm has been demonstrated to be convergent and stable [1].

We will use this task as an example to illustrate our unified modelling approach. The present section details the first step: a microscopic simulation of the problem [1]. The simulations are implemented using the multi-agent framework Flame. The software allows us to set up any number of individual agents whose behaviour is specified by user-defined subroutines. Initially, 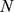 robots are distributed over a compuational domain with side length 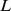. Each robot has a certain probability 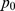 to start as “red'' and initial velocity 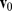. The magnitude of the velocity 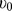 is fixed as a simulation parameter and the spatial orientation is randomly chosen using a uniform probability distribution. After updating its position according to the current velocity 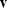, the robots broadcast their current position and check if any number of robots is inside their avoidance radius 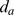. If this is the case, the robot turns by 180 degrees, in order to avoid double counting of robots, and adds the color of the encountered robot to an internal list. Note that, in reality, mechanical effects in the actuators will afflict the heading with a certain error (compare subsection 4). We performed runs with a varying degree of inaccuracy and checked that the results are not affected as long as there is no preferred direction. Hence we only present simulations without error in the heading. If the total number of encounters equals 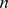, the robot changes its own color according to the majority of encounters. The simulation parameters are given in Table 1. These parameters are chosen to allow an easy comparison with the results of Hamann *et al.* [1].

**Table 1:** Parameters for the microscopic simulation of the virtual swarm using Flame.

Speed of agents 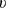	0.01 [length units/time units]
Number of agents 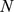	150
Initial prob. to be "red'' 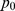	0.5
Avoidance radius 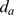	0.01 [length units]
side length of domain 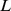	1 [length units]
run time 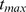	5000 [time units]
number of simulations 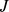	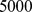

### 2 The symmetry parameter

To describe the macroscopic behaviour, we define a (discrete) symmetry parameter 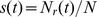 with 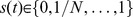, where 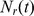 denotes the number of red agents at time 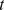 [1], [19]. The term symmetry parameter indicates the similarities to the order parameter in statistical physics. The order parameter is used to describe phase transitions in statistical mechanics. Likewise, the symmetry parameter follows the progress from an unordered system to an ordered state. Due to our simulation setup, the probability distribution function (PDF) 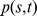 will initially be binomial, 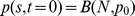. With time, the system evolves into a steady state where 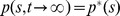 will be bimodal with peaks at 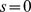 and 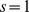. Contrary to intuition, the states 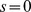 and 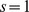 are not absorbing: even though there are no collision partners of the opposite color available: some agents might still have stored a majority of opposite-color encounters in their memory and will change their own colour at a future encounter. However, the simulations will show that, to a good approximation, no fluctuations occur at the boundaries. Eventually, we expect all agents to agree on one color.

The evolution of the symmetry parameter has been described as a Markov process [1]. We will show in Section “Constructing a time coarse-grained Markov process for the symmetry parameter'' that, in general, the time evolution of 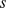 is not Markovian. This is intuitively clear since the robots keep track of their previous encounters and, as such, the process cannot be memoryless. However, we argue in Section “Constructing a time coarse-grained Markov process for the symmetry parameter'', that a Markov process can be constructed on coarse time scales which are larger than the typical time between individual encounters. Hamann *et al.* do exactly this [1], but do not present such an argument. Instead they justify their assumption empirically through numerical experiments. For now, we just assume that such a process exists and is a reasonably good approximation of the behaviour of 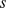 over time.

Obviously, 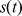 can only assume discrete values and hence the corresponding stochastic process should be discrete. However, for large 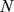, the separation between the discrete levels of 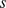 becomes negligible and a continuous approximation can be employed [1]. 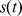 then obeys an Ito stochastic differential equation (SDE) of the type 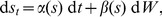
(1)


where as usual 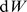 denotes the differential of the Wiener process and the parameters 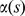 and 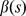 need to be determined from the simulation output as follows. The equivalent Fokker-Planck equation (FPE) is given by 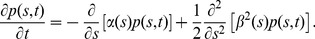
(2)


**Figure 1:**
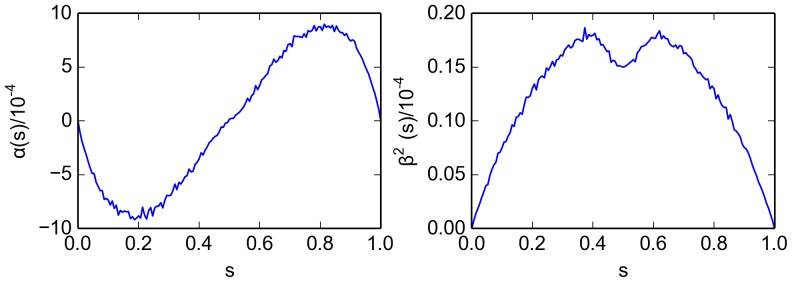
Parameters 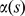 (left) and 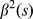 (right) for the stochastic differential equation as inferred from the simulation results for the virtual swarm. Simulation parameters are given in Table 1.

We extract the information about the macroscopic continuous-time process from the simulation output (details can be found in Appendix 1.1). The process is time homogeneous and we can use the whole time interval to compute the SDE coefficients. 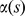 and 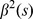 for the model in Table 1 are shown as a function of 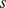 in Fig. 1. The deterministic component 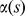 (left panel in the figure) acts to promote an emerging decision. For example, if more “red'' robots are present (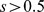), the drift component will further push the symmetry parameter towards 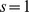. The diffusivity 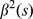 attains its maximum if a decision is emerging but still enough robots of the opposite color are present to revise that decision. 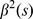 exhibits a local minimum at 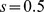 where fluctuations of opposite directions tend to cancel each other out. It is clear from the figure that the boundary fluctations (at 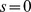 and 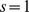) are negligible. Note that Hamann *et al.* include a probabilistic decision error in their model [1]. If robots are allowed to make an error with a certain probability when changing their color, a non-vanishing deterministic component 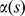 at 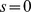 and 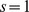 will appear [compare Fig. 1.d in [1]].

**Figure 2:**
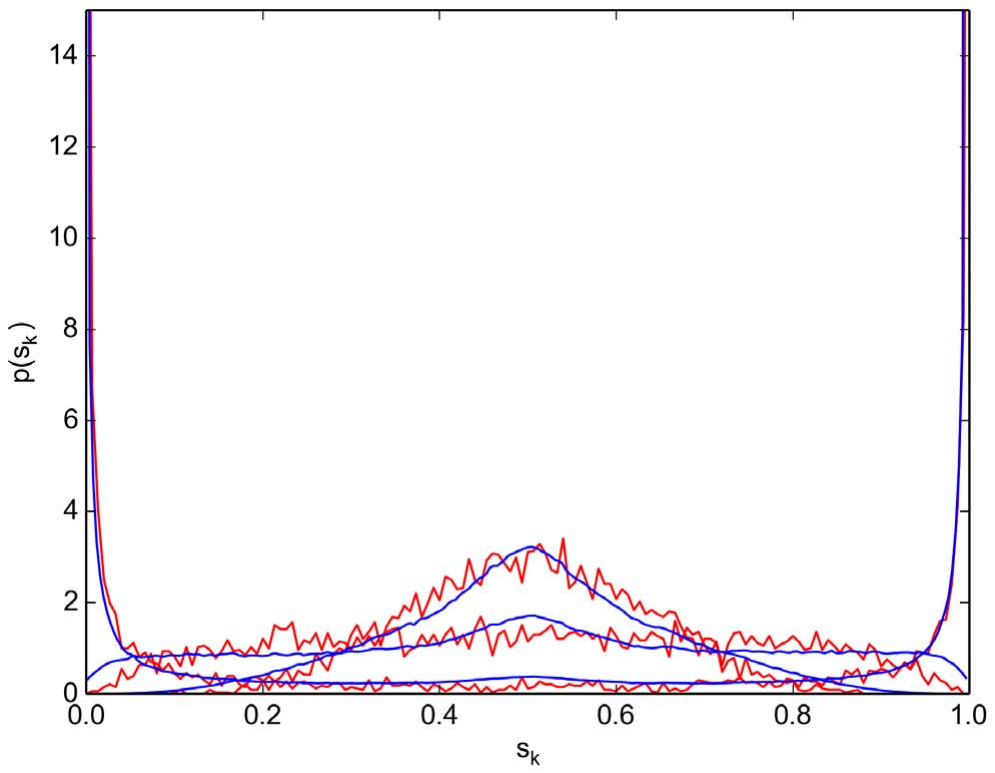
Probability density function. Probability density function 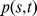 as obtained from the microscopic simulation (red curve) and from the numerical integration of the FPE [Eq. (2)] (blue curve) at times 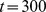 s, 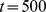 s and 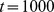 s. The FPE coefficients were constructed from the microscopic simulation of the virtual swarm (cf. Fig. 1).

We integrate Eq. (2) numerically, using the previously obtained 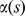 and 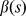, with an initial binomial distribution centered at 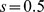


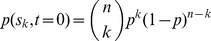
(3)


where 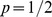 and the index 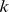 on 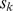 indicates the discrete range of 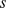 such that 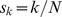 with 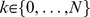. The numerical integration was performed using the simulation package inchman (http://inchman.github.io/Inchman/) with a vanishing probability flux at the boundaries. Fig. 2 displays 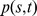 at three different times (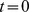 s, 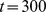 s and 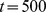 s) from the simulation results (red curve) and from numerical integration of Eq. (2) (blue curve).

### 3 Stationary distribution, splitting probability, and time to decision

Following Hamann *et al.* [1], we consider the splitting probability 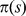 as a measure for the robustness of the process. 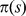 is defined as follows. Consider the stochastic process (1) with two absorbing boundaries erected at 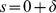 and 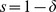, where 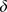 is termed the decision threshold. The splitting probability 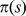 is then defined as the probability that, if the process starts at 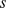 at 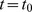, it will first exit through the boundary 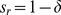. In other words, 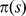 is the probability that a system starting in the state 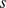 will come to the majority decision “red''. We also introduced the steady state distribution 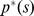, which is defined as the probability to find the process in the state 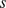 after it has attained its stationary state. For all simulations presented in this article, this was the case for 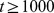. Appendix 1.2 demonstrates how 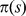 and 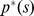 can be computed from the simulation output.

**Figure 3:**
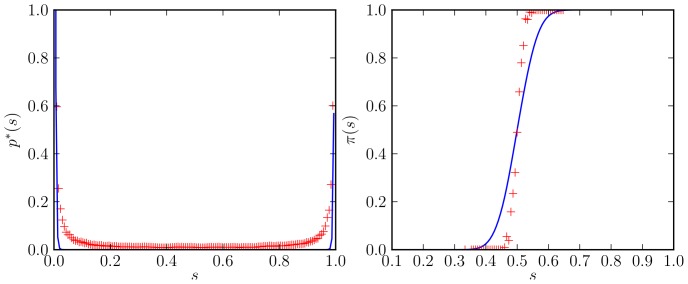
Stationary probability distribution and splitting probability for a virtual swarm. (left) Stationary probability distribution 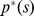 as estimated from the simulation output (red markers) and computed from the FPE coefficients [Eq. (56) in Appendix 1.2]. (right) Splitting probability 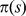, as estimated directly from the simulation output [cf. Eq. (53) in Appendix 1.2] (red markers) and computed using the integrated stationary distribution [Eq. (54) in Appendix 1.2] (blue curve). Simulation parameters are given in Table 1.

In Fig. 3, we compare the steady state distribution 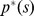 (left panel) and 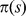 (right panel) obtained from the simulation results [Eq. (53) in Appendix 1.2], plotted as red markers, and the splitting probability computed through the FPE [Eq. (54) in Appendix 1.2] (blue curve). The stationary PDF from the simulation results (red markers in the left panel) is small but non-vanishing in the interior region and smoothly rises at the boundaries to meet the bimodal behaviour of the FPE solution (blue curve). This is expected since we only use a limited time interval from the simulation output to compute 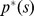. We expect that the agreement will be the better the longer the time interval is. The shape of the splitting probability distribution from the simulation results (red markers in the right panel) strongly resembles the step function with a sharp rise at 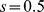. This behaviour corresponds to a low decision error: if the system starts in a state with a majority of “red'' robots (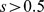) it will almost always find the consensus “red'' (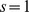) and vice versa. In contrast, the integrated FPE exhibits a comparably smooth transition centered at 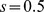 with a considerable decision error. While it is also difficult to achieve a precise numerical intergation of this FPE, we can identify at least two systematic reasons for these deviations. First, the underlying stochastic process for 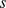 is not Markovian (see Sec. 2). Second, by only considering the spatially-integrated variable 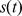, we implicitly disregard any influence spatial variations might have. We will return to these issues in Sections “The macroscopic-discrete approach'' and “Constructing a time coarse-grained Markov process for the symmetry parameter'', respectively, and fully analyze their influence. For now, we note that robots of the same color tend to form clusters that are hard to break up and hence promote an emerging decision. Even if the spatial variability of the system is comparably low (see Section 4) we expect that cluster formation improves the decision accuracy.

**Figure 4:**
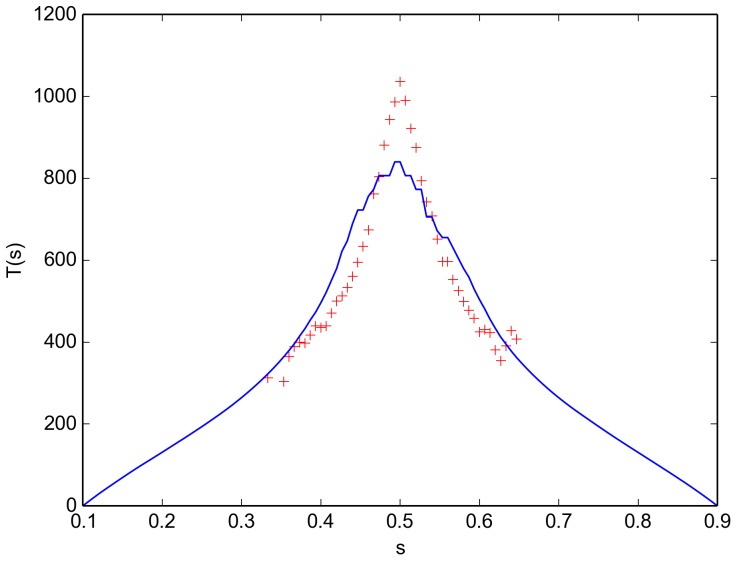
Decision time for a virtual swarm. Decision time 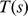 as estimated from the simulation output [Eq. 58] (red markers) and computed from the FPE coefficients [Eq. (59)]. Simulation parameters are given in table 1.

The last swarm property that we compute is the average time to decision (see Appendix 1.2 for details). We display a comparison between the decision time as computed directly from the simulation output (red markers) and from integrating the FPE (blue curve) in Fig. 4. For systems that start far away from the center, 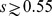 or 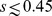, we have a good agreement between the FPE solution and the simulation results. If the system is prepared in a state with an approximately equal number of robots of each color, the FPE solution is about 25 per cent lower than the actual simulation solution. It appears that our description underestimates the stochastic variance in 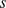 close to the center. We do note, however, that the first passage time generally has a high standard deviation and varies over many orders of magnitude with varying noise levels [20].

### 4 Equations of motion and spatial correlations

In the original setting [1], the robots move in a straight line, with a constant velocity 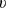, until they encounter another robot. Upon collision, the robot simply reverses its direction. In reality, both the position as well as the heading, i.e. the direction of 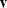, will be subject to some variation. These effects will introduce a certain amount of randomness into an otherwise deterministic system. To take this into account, we describe the motion of a single robot in terms of a continuous-time correlated random walk, more specifically a velocity-jump process [21]. Here, collisions between single robots are modelled as a Poisson process of intensity 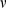. At each collision, the robot changes its heading by an angle 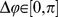, which is chosen from a Gaussian distribution 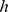 centered at 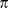: 
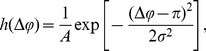
(4)


where the normalization factor 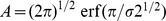 is chosen such that 
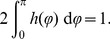
(5)


It can be demonstrated that such a system behaves, after a transitional period, approximately diffusive [21]. For our purpose, however, it is sufficient to know that the distribution of the robot headings remains isotropic under the assumption that it is isotropic initially. We can see that as follows. The probability to find a particular velocity 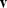 after a collision, provided the current velocity is 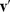, is given by the reorientation kernel 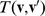 which depends on 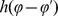 as 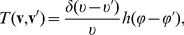
(6)


where 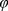, 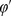 denote the polar angles, i.e. the headings, of 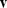 and 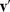, respectively, and the Dirac delta distribution 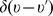 indicates that the robot speed is conserved throughout the collision. Clearly, if the initial distribution of headings is uniform, 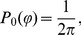
(7)


the probability to find a particular 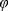 heading after the collision is given by 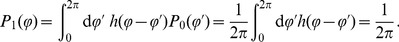
(8)


Thus, 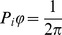 for all 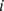. Not surprisingly, if the headings are initially isotropically distributed, they will remain so during the course of the simulation. This observation justifies our assumption that the size of the velocity error does not affect the validity of the time-continuous Markov model and we consequently set it to zero. In this limit, the reorientation kernel approaches the Dirac delta distribution in the velocity angle 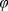.

When deriving a probabilistic macroscopic description from microscopic principles, authors often assume a homogeneous distribution of individual agents [8], [9]. It is, however, not immediately clear that such an assumption would be justified in all cases. A first estimate of how homogeneously distributed our system is can be obtained by comparing the characteristic length scale to the linear dimensions of the compartment [22]. The characteristic length scale in our case is given by the mean free path between subsequent collisions, 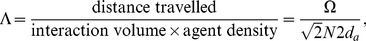
(9)


where 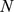 is the total number of agents in the integration domain of size 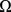 and 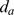 denotes the avoidance radius. If 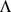 is of the same magnitude as the linear dimensions of the compartment, we expect that all density fluctuations are of larger scale than the compartment dimensions and hence we can assume a homogeneous distribution. In particular, Eq. (9) states that homogeneity is better satisfied for smaller avoidance radii 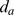.

For our microscopic simulations, direct information about the spatial distribution is available and we can use this information to estimate the degree of spatial correlation. From the multitude of available second-order characteristics for point processes, we here opt for the pair correlation function 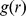, which is popular throughout a wide variety of disciplines ranging from astrophysics to biology [23]. Heuristically, 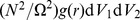 gives the probability to find two individuals in the infinitesimal volumes 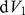 and 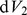, which are separated by a distance 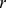. In writing down 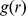, we employ the fact, which we established above, that our system does not exhibit a preferred direction, i.e. it is isotropic, and all spatial correlations can only depend on the absolute value of the separation 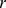. We can compute 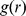 directly from the simulation output (Appendix 1.3).

**Figure 5:**
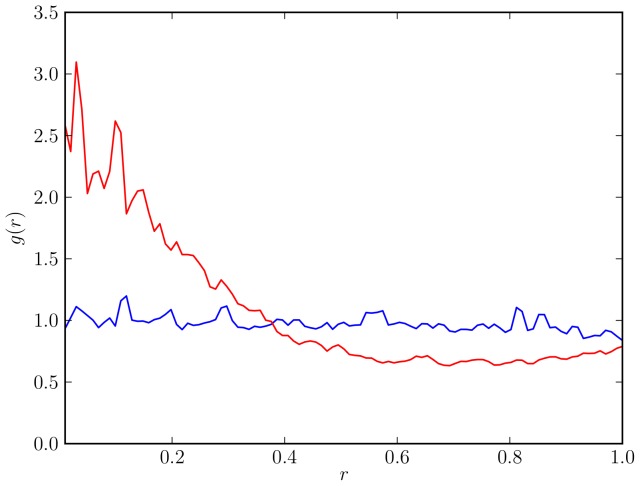
Pair correlation function for a virtual swarm. Comparison of the pair correlation function 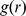 [Eq. (60)] for different values of the avoidance radius 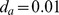 (blue curve) and 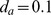 (red curve). All other simulation parameters are given in table 1. For a large avoidance radius (red curve), corresponding to 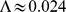 [Eq. (9)] the system tends to form spatial clusters. In contrast, if the avoidance radius is low (blue curve, 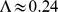) the distribution is nearly homogeneous.

The pair correlation function 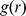 allows us to estimate the degree of clustering exhibited by the stochastic system. For a Poisson, i.e. homogeneously-distributed, point process, 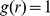. If the process forms clusters at a particular scale 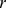, 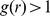, and, likewise, 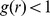 if it avoids this scale, i.e. fewer agents are found at this distance than would be expected if the process was homogeneously distributed [23]. In Fig. 5, we compare 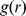 for a virtual swarm using two different avoidance radii 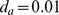 (blue curve) and 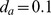 (red curve). It is immediately obvious that the process with 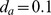 (red) exhibits a large clustering at small length scale and, consequently, we can expect that spatial structures will be important in this case. On the other hand, for small 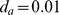 (blue curve), the “red” agents are approximately homogeneously distributed. Fig. 5 substantiates our back of the envelope estimate Eq. (9), since 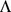 is inversely proportional to the avoidance radius. For 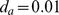, the characteristic length scale evaluates to 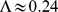 which is roughly of the order of the linear dimensions. Conversely, for 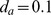, we find 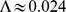, which is one order of magnitude smaller than 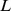.

The clustering at high 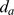 can be explaind as follows. Essentially, 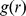 is the probability (modulo the normalization factor) to find two red robots at two randomly-picked spots (separated by a distance 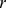) of the total area. 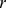 is then the distance between these two robots and if 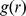 is higher than one, it means that robots seem to be more likely to be found at that distance than one would expect for a spatially-homoheneous distribution. Note that robots can indeed be closer than the avoidance radius. If 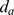 is large and the number of agents is high, the total avoidance area exceeds the integration area and robots have no chance to steer clear of other robots. In fact, while being able to communicate over a wider range, they remain relatively stationary. This explain the large clustering in this limiting case. The immobility of the robots prevents clusters from being disrupted through robot movement, which is consistent with the concept of the mean free path.

### 5 Avoidance radius

**Figure 6:**
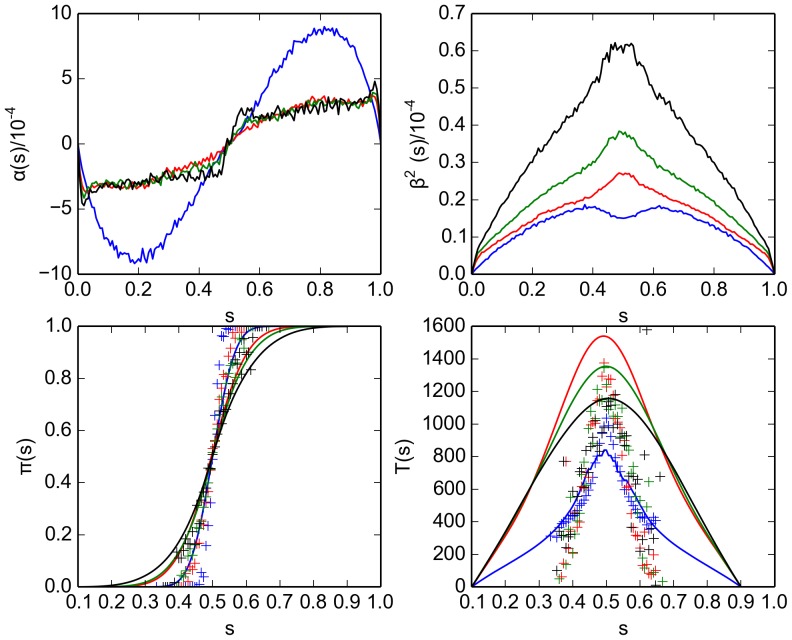
Comparison of swarm properties for different values of the avoidance radius 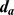. Shown are results for 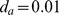 (blue curves and markers), 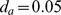 (red curves and markers), 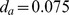 (green curves and markers) and 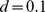 (black curves and markers). All other simulation parameters are given in Table 1. (top panels) Parameters 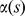 (left) and 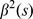 (right) for the stochastic differential equation as inferred from the simulation results for the virtual swarm. (bottom left panel) Splitting probability 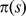, as estimated directly from the simulation output [cf. Eq. (53)] (markers) and computed using the integrated stationary distribution Eq. (54) (curves). (bottom right panel) Decision time 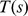 as estimated from the simulation output [Eq. 58] (markers) and computed from the FPE coefficients [Eq. (59)] (curves).

In order to investigate how spatial inhomogeneities affect the validity of our approach, we perform numerical experiments where we vary 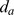 over a range of values. In Fig. 6, we compare swarm properties for 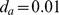 (blue curves and markers), 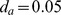 (red curves and markers), 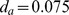 (green curves and markers) and 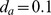 (black curves and markers). All other simulation parameters are as in Table 1. Shown are the SDE coefficients (top panels), the splitting probability (bottom left panel) and the decision time (bottom right panel).

We first notice that for a large avoidance radius (black), the drift 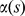 (top-left panel) becomes very flat with a sharp rise at 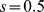 and almost resembles a step function, which means that the drift parameter 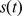 will move towards a decision with almost constant, albeit low, speed. This is clearly an effect of spatial clustering: only the cluster members that are located at the boundary can convince other robots to change their decision. In contrast, in the homogeneous case, each robot of the majority color has a large chance to encounter a robot of the opposite color. Hence, the probability for a random agent to change its color is relatively high when a particular color is already prevailing. This explains the large drift in this case (top-left panel, blue curve).


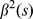
 (top right panel) has a distinct peak in the inhomogeneous case (black curve) at 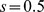. 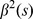 represents the variability of the agent colors and it is high when agents tend to frequently revise their decisions. This is the case in the early cluster-formation stage of the system evolution (at 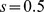). Once the clusters have formed, color changes occur less frequently and in a more systematic manner, hence 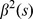 declines towards 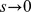 and 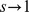. In the homogeneous case (blue curve), the change rate is comparably low at 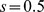: any particular robot will, with approximately the same probability, encounter robots of the same and of the opposite color and hence is not very likely to change its color. The situation is different, when one color is slightly dominant (at 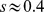 and 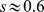). In this case, there are still a lot of agents of the non-dominant color available. However, these robots are more likely to encounter a robot of the dominant color, i.e. opposite, color and change occurs. This explains the double peak in the blue curve. Finally, 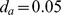 (red curve) is the limiting case, where the total avoidance area is comparable to the stage area and clustering becomes dominant. Here, the double peak has vanished and a distinct single peak becomes visible.

Finally, the wide shape of the decision time curve (bottom-right panel) follows immediately from the large diffusivity in the high and low 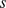 regions. In contrast, the actual decision time (black markers in bottom-right panel) is low: if the system is prepared in a state with a large majority, a consensus is achieved quickly. The decision time curve also illustrates the qualitative change that occurs in the transition from the low spatial correlation regime (blue curve) to the regime where clustering becomes dominant (red, blue, and black curves). Experiments with a low avoidance radius 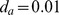 where no clustering occurs (blue curve) are characterized by a high drift contribution (compare top-left panel and discussion above) which leads to a low decision time. In contrast, clusters tend to be stable and spatial correlations greatly increase the decision time. The same effects can be observed in the splitting probability plot (bottom-left panel). Clustering might lead to wrong decisions as large stable clusters can overturn a developing decision.

In summary, clustering, when it occurs, has a large impact on the shape of the SDE coefficients. The change is qualitative (compare the blue and the black curves in the top panels) and can be likened to a phase transition of the macroscopic system. Naturally, if spatial effects dominate, our homogeneous description loses its validity. We conclude that our approach is best-suited for small avoidance radii, when the system is approximately homogeneous.

## Kilobot Experiments

Simulations, by their very nature, can only model the swarm behaviour in very idealized conditions. It is impossible to incorporate all real-world challenges, such as changing environmental conditions and noisy sensors and actuators, into a simulation model *a priori*. One possibility to achieve a better understanding of the actual physical swarm behaviour is to perform experiments under controlled conditions in a laboratory setting [4]. Here, we present results from experiments we conducted using Kilobots.

### 1 Experiment setup

Kilobots are low-cost robots specifically designed to enable large-scale experiments on swarm behaviour [16]. Locomotion of the Kilobots is achieved using vibration motors which allows them to move at about 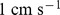 and change their heading at an angular velocity of 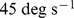. Kilobots can communicate via infrared light, which is emitted isotropically by a LED at the bottom of the Kilobots. Hence, communication requires the use of a reflective table that allows another robot to detect the reflected signal. The maximal communication distance is about 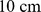. Measuring the light intensity allows distance estimates between two Kilobots with a maximal accuracy of 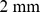.

**Figure 7:**
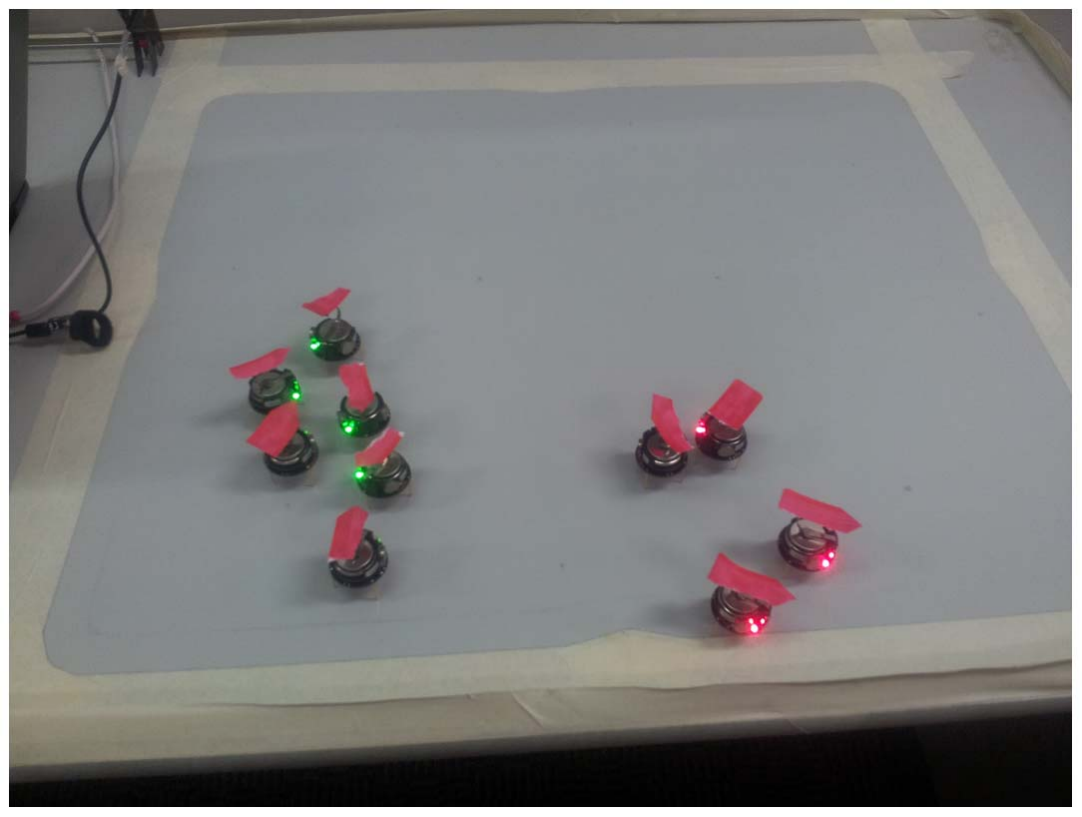
Setup for the kilobot experiments. The arrow marks the current heading of each bot.

**Figure 8:**
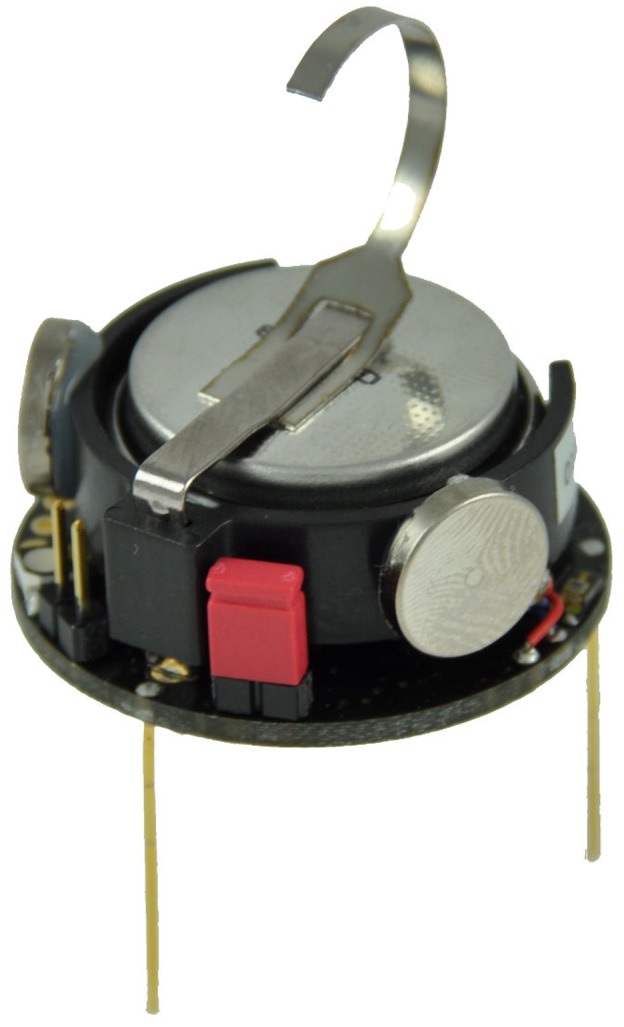
Picture of a kilobot. Picture courtesy of K-Team (http://www.k-team.com/).

For our experiments, we used a swarm of ten Kilobots moving freely on a flat table. The Kilobots are confined to a square area of 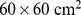 which was marked out using layered tape. Initially the Kilobots were distributed homogeneously over the table such that they are outside the communication distance of each other. Each Kilobot is initialized to either “red'' or “green'' state, with a probability of 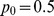. The state of each bot is indicated by a red or green LED. The Kilobots achieve consensus using a behavioural algorithm similar to the density estimation algorithm presented above [24]. The communication distance is transmitted using an integer value 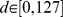. By experiment, we found that 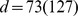 corresponds to 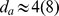 cm and we assume that inside this range the relationship is linear. Fig. 7 illustrates the basic setup. An actual Kilobot is shown in Fig. 8. The swarm parameters are given in Table 2.

**Table 2:** Parameters for the kilobot swarm and the microscopic simulation of it using Flame.

Speed of agents 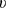	1 [ 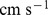 ]
Number of agents 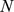	10
Initial prob. to be "red'' 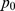	0.5
Avoidance radius 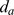	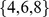 [cm]
side length of domain 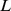	60 [cm]
run time 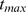	2000 [s]
number of simulations 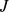	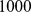

### 2 Results

We perform experiments where we vary over the communication distance (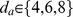 cm). Note that the communication distance of the Kilobot is exactly the avoidance distance of the virtual swarm. For each value of 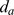, we conduct 31 experiments and record the initial number of “red'' robots and the time stamps when a robot changes its color. The experiments were run until consensus is achieved. In principle, we could extract all swarm parameters from the experiment records as in Section “Microscopic approach: Virtual swarm'' (subsection 3). However, given the small number of robots, it is questionable if the Fokker-Planck approximation [Eq. (2)] of the underlying continuous-time Markov chain is still applicable. We will return to this question in Section “Constructing a time coarse-grained Markov process for the symmetry parameter''. For now, we elect to model the time evolution of the symmetry parameter as a continuous-time, discrete-state Markov process 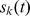 (where the index 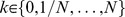 indicates the discrete nature of the process) with the corresponding Master equation [20]

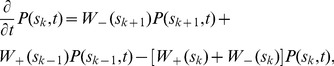
(10)


where the stepping functions 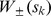 are defined such that 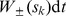 gives the probability for the process to jump into the state 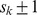 in the next infinitesimal interval 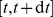, given that it is currently in the state 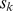. From the definition of 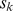 as the proportion of robots to be in state “red'', it follows that 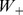 denotes a change in color from “green'' to “red''. We expect that the continuous-state FPE description [Eq. (2)] naturally follows from Eq. (10) in the limit 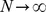 if a suitable limiting procedure, such as the system-size expansion [25] is applied. This result is demonstrated in Section “Constructing a time coarse-grained Markov process for the symmetry parameter'' for a simplified toy problem.

We can easily estimate the jump probabilities 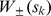, the splitting probabilities 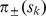 and the average time to decision 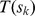 from the experiment records using standard techniques [20]. For convenience, we collect the main literature results in Appendix 1.4.

**Figure 9:**
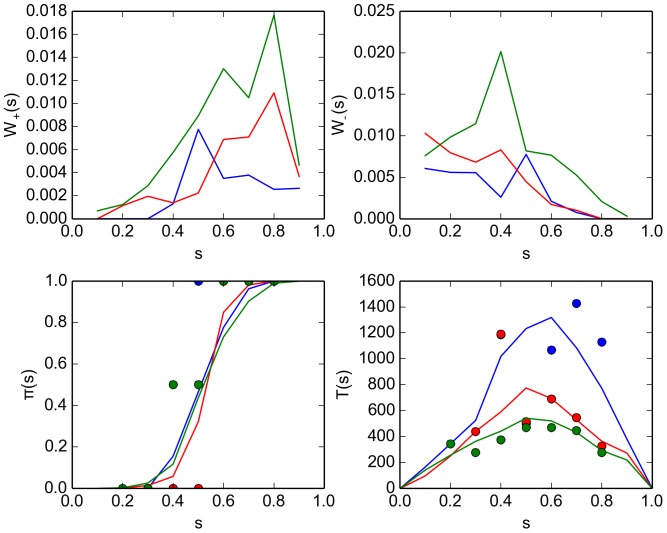
Comparison of properties of the kilobot swarm for different values of the avoidance radius 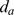. Shown are results for 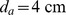 (blue curves and markers), 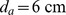 (red curves and markers) and 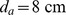 (green curve and markers). The experimental setup is described in Section "Kilobot experiments'' (subsection 1). (top panels) Jump probabilities 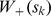 (left) and 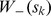 (right) for the Master equation (10) as inferred from the experiment records [Eq. (63)]. (bottom left panel) Splitting probability 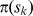, as estimated directly from the experimental records [cf. Eq. (53)] (markers) and computed using the solution of the Master equation Eq. (68) (curves). (bottom right panel) Decision time 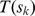 as estimated from the experiment records [Eq. 58] (markers) and computed from the Master equation [Eq. (69)] (curves).

The results are presented in Fig. 9, which displays the jump probabilities 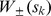 (top panels), the splitting probability 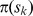 (bottom left panel) and the decision time 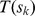 (bottom right panel) for avoidance distances 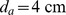 (blue), 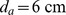 (red), and 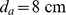 (green). In the bottom panels, the markers indicate the results extracted directly from the experiment records while the curves are computed from the solution of the Master equation (10). Generally, the variance is high and a clear trend is hard to recognize in the plot. This is expected, as the number of experiments we performed is low (31 experiments for each 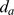). The jump probabilities exhibit a tendency to promote an emerging decision: if a majority of “red'' robots is present (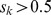), 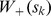 is high and the process will further move towards the consensus “red'' (and vice versa).

Given the high variance of the transition probability, the agreement between the solution of the Master equation (curves in bottom panels) and the experimental results (markers in bottom panels) is reasonable for the average decision time. However, for the splitting probability the computed solution is quite poor. As in Section “Microscopic approach'' (cf. Fig. 6), the integrated solution appears to overestimate the decision error, i.e. the integrated solution suggests that a system prepared in a state close to 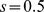 is likely to make a wrong decision. This disagrees with the experiment where wrong decisions are virtually non-existent. One possible cause for this is that the low number of experiments caused a high variance in the transition probabilities and that solving the Master equation with these yields inaccurate results. Much more importantly, we can identify a systematic reason why this might be the case: Projecting the multi-dimensional configuration space of the underlying problem, which consists of the current position and collision history of each robot, onto a one-dimensional finite space, the symmetry parameter, requires spatial homogeneity as a necessary (but as we will demonstrate in Sections “The macroscopic-discrete approach'' and “Constructing a time coarse-grained Markov process for the symmetry parameter'' not sufficient) condition. In Section “Microscopic approach: Virtual swarm'' (subsection 4) above, we identified the mean free path betweeen collisions 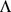 [Eq. (9)] as the characteristic length scale of spatial inhomogeneities. For this setup, we find 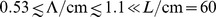, i.e. we expect distinct clustering of robots of the same color. Indeed, Fig. 7 clearly shows the formation of two clusters.

**Figure 10:**
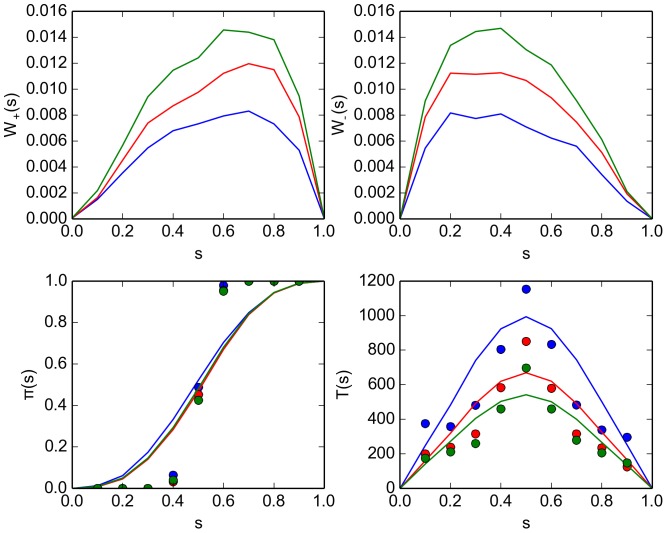
Comparison of properties of a flame simulation designed to mimic the kilobot swarm for different values of the avoidance radius 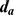. Also compare Fig. 9). Shown are results for 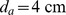 (blue curves and markers), 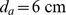 (red curves and markers) and 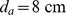 (green curve and markers). The experimental setup is described in Section "Kilobot experiments'' (subsection 1). (top panels) Jump probabilities 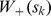 (left) and 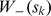 (right) for the Master equation (10) as inferred from the experiment records [Eq. (63)]. (bottom left panel) Splitting probability 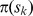, as estimated directly from the experimental records [cf. Eq. (53)] (markers) and computed using the solution of the Master equation Eq. (68) (curves). (bottom right panel) Decision time 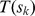 as estimated from the experiment records [Eq. 58] (markers) and computed from the Master equation [Eq. (69)] (curves). The simulation parameters are given in table 2.

For comparison, we use flame to simulate a swarm with the same parameters as the kilobot swarm (Table 2). The results are presented in Figure 10. The splitting probability 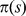 (bottom left panel in the figure) exhibits the same mismatch between the simulation data (markers) and the integrated solution (curve) despite the fact that the number of experiments is significantly higher (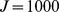). Again, we attribute the discrepancy to spatial correlations (see subsection 5 in Section “Microscopic approach: Virtual swarm'').

## The Macroscopic-Discrete Approach

An important aim of this work is to explore how well self-organizing phenomena can be represented using the mathematical tool box of stochastic chemical kinetics [26]. The standard approach to formulate individual-level behaviour uses Langevin-type stochastic differential equations to describe the motion and interaction of individual agents. A *macroscopic-discrete* description, however, only keeps track of the number count of a population of identical and indistinguishable individuals, termed *species*, in a spatially-homogeneous integration domain.

For the task at hand, we introduce two species classes, namely 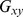 and 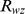. The capital letter denotes the color of the agent (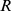 for red and 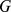 for green) while the subscripts 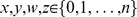 stand for the number of previous encounters of “red'' (first indices 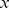 and 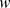) and “green'' (second indices 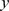 and 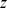) robots. For example, an individual of species 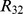 is of color “red'' and has previously encountered three “red'' and two “green'' robots. We model the interactions, i.e. the collision encounters, using the following set of reactions: 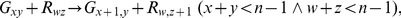
(11)

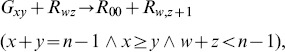
(12)

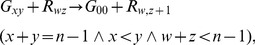
(13)

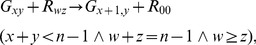
(14)

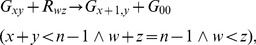
(15)

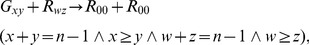
(16)

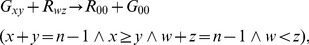
(17)

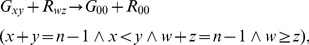
(18)

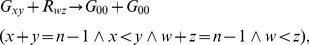
(19)


and corresponding sets for interaction between equal colors. For 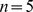, we find a total of 30 species and 465 reactions. A decision depth of 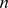 yields 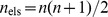 elements per species and a total of 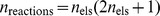.

In a reaction network the time evolution of the population count for the various species can be described by the well-known chemical Master equation (CME) and a large arsenal of analytical and numerical methods for the solution of the CME is available [27]. In particular, very efficient Monte-Carlo methods exist to numerically compute individual realizations of the underlying stochastic distribution [28]. A macroscopic-discrete model assumes that the various components (species) are well mixed in the container, i.e. spatial homogeneity of the system. We established in Section “Microscopic approach: Virtual swarm'' (subsection 4), that the well-mixed constraint is satisfied, if the mean free path between collisions is at least comparable to the container dimensions. Here and in the following, we assume that the system is spatially homogeneous. In this section, we present results of macroscopic-discrete simulations of the robot swarm and investigate how well the microscopic model (Section “Microscopic approach'') is approximated by a macroscopic-discrete description. In the next section (Section “Constructing a time coarse-grained Markov process for the symmetry parameter'') we make an attempt at analytically solving the chemical Master equation.

### 1 Deriving the reaction constants from the avoidance radius

The density estimation algorithm as described previously [1], [24] permits communication between individual robots only if the distance 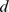 between the individuals is below a certain communication or avoidance distance 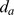. In the language of chemical kinetics, this communication distance corresponds to the reaction radius 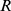 in the sense that interaction reactions [Eqs. (11)–(19)] occur if the distance between the individual “molecules'' 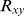 and 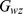 is smaller than 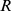. The reaction is deterministic in the sense that it will always occur if the molecules are close enough. This is different to chemical kinetics, where molecules react only with a certain probability, which depends on the particular reaction mechanism. The macroscopic-discrete model, on the other hand, assumes that the reaction propensity per compartment is given by the law of mass action, viz. 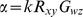, where 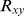 and 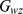 denote the number of particles of each species in this compartment.

The question of howto translate 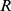 into the rate constant 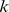 for the bi-molecular reactions (11)–(19) has a long-going history in the field of chemical kinetics. For our purpose, it suffices to work in the “ballistic regime'', where the trajectories of particles (or robots, in our case) between collisions are essentially straight lines [29], [30]. It can then be shown that the reaction constant is given by [30]

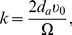
(20)


where 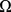 is the area of the compartment. This is the encounter rate, when has also been used in previous work by other authors [31], [32]. Note that, in chemical kinetics, the actual reaction rate might be lower as the encounter rate quoted here, since reactions might only occur with a certain probability upon encounter. The derivation hinges on an isotropically-distributed robot velocity and assumes that the robots are homogeneously distributed inside the compartment. We demonstrated above (subsection 4 in Section “Microscopic approach: Virtual swarm'') that an isotropic distribution of the robot headings is guaranteed as long as the robot velocity is isotropic initially.

### 2 Macroscopic-Discrete simulations

We perform simulations of the reaction network Eqs. (11)–(19) using the highly efficient, GPU-accelerated, simulation package inchman. Inchman allows us to perform a large number of Monte-Carlo experiments of the reaction network using an accelerated implementation of the classical direct method [17], [18], [33]. We set up simulations designed to reproduce the virtual swarm (Section “Microscopic approach''). The species and reaction between species are defined as in Eqs. (11)–(19) and we set up the reaction rate according to Eq. (20), where the reaction radius 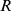 is given by the avoidance radius 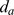. The simulation parameters are given in Table 3. The large number of experiments (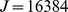) is only feasible with an accelerated macroscopic-discrete simulation.

**Table 3:** Parameters for the macroscopic-discrete simulations of the virtual swarm using inchman.

Speed of agents 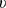	0.01 [length units/time units]
Number of agents 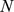	150
Initial prob. to be "red'' 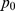	0.5
Avoidance radius 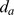	0.01 [length units]
side length of domain 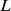	1 [length units]
run time 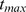	4000 [time units]
number of simulations 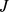	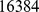

Parameters for the macroscopic-discrete simulations of the virtual swarm using inchman. The reaction rate is given by Eq. (20).

**Figure 11:**
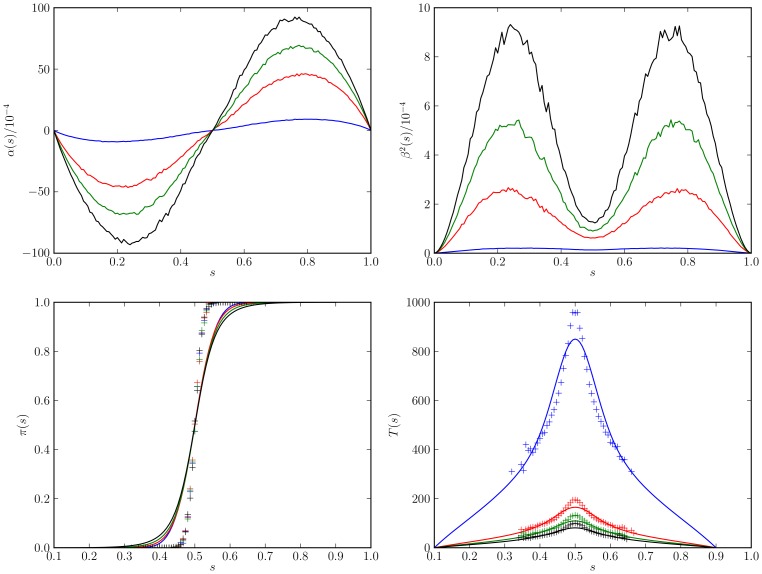
Comparison of swarm properties for macroscopic-discrete simulations of the virtual swarm using different values of the avoidance radius 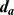. Shown are results for 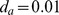 (blue curves and markers), 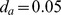 (red curves and markers), 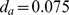 (green curves and markers) and 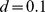 (black curves and markers). All other simulation parameters are given in table 3. (top panels) Parameters 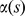 (left) and 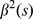 (right) for the stochastic differential equation as inferred from the simulation results for the virtual swarm. (bottom left panel) Splitting probability 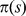, as estimated directly from the simulation output [cf. Eq. (53)] (markers) and computed using the integrated stationary distribution Eq. (54) (curves). (bottom right panel) Decision time 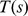 as estimated from the simulation output [Eq. 58] (markers) and computed from the FPE coefficients [Eq. (59)] (curves).

The results of these experiments are presented in Fig. 11. On comparison with the microscopic simulation, Fig. 6, we note that the SDE coefficients (top panels in both figures), exhibit the same characteristic shape for a low avoidance radius (blue curve). The drift function (top left panels) acts to promote an emerging decision while the diffusivity (top right panels), which peaks at the same values of the symmetry parameter as the drift function, allow emerging decisions to be revised. However, the peak value of 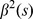 (at about 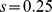 and 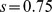) for the macroscopic-discrete simulations (Fig. 11) is higher while 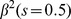 is roughly the same for the macroscopic-discrete and the microscopic simulations. This behaviour can be explained as follows. In the macroscopic-discrete simulations, we explicitly disregard any spatial correlations such as clustering - which we know (subsection 4 in Section “Microscopic approach: Virtual swarm'') are prevalent for high 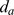. Consequently, the SDE coefficients (top panels) are accurately reproduced for low 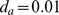 (blue curves) while they markedly differ for higher radii. The large avoidance radius case (for example black curves for 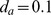) is basically a scaled version of the homogeneous case. The scaling is due to the higher reactivity 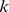, which stems from the larger 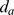 [compare Eq. (20)]. The lower panels in the figure demonstrate that the macroscopic-discrete approach is consistent in that the swarm properties splitting probability (bottom-left panel) and decision time (bottom-right panel) are fairly well reproduced by the FPE solution (curves) for all 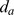.

## Constructing a Time Coarse-Grained Markov Process for the Symmetry Parameter

We have shown in the previous sections how the time evolution of the symmetry parameter 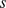 can be described using a stochastic differential equation [Eq. (1)] and how the coefficients of this SDE can be extracted from the results of numerical and physical experiments (Sections “Microscopic approach'' and “Kilobot experiments''). Inspired by results from chemical kinetics, we could also demonstrate in Section “The macroscopic-discrete approach'' how the microscopic description of the robot swarm gives rise to a macroscopic-discrete description which focuses on aggregate properties of the swarm. What is left open is the important question how to determine the SDE coefficients 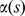 and 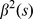 analytically. In this section, we close the loop by outlining a new way to derive 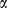 and 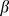 analytically from the macroscopic-discrete description. Again we will refer to techniques from chemical kinetics as our task amounts to solving the chemical Master equation for the reaction network given by Eqs. (11)–(19). Unfortunately, even for the lowest sensible decision threshold (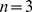), the sheer size of the reaction network (78 reactions) makes such an attempt a daunting undertaking. We therefore opt to postpone a full solution of the problem and instead tackle a very simplified toy problem, hoping to gain valuable insights into the structure of the solution. The results presented in this section are therefore only to be seen as an in-principle investigation that paves the way towards a complete solution.

Our toy problem consists of two species, “red'' and “green'', that can be in two states each, a ground state 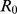 and an excited state 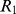 (and analogous for green). Whenever an individual of species, say “red'', in the ground state encounters any “green'' individual (in the ground state or in the excited state), it changes into the excited state 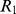. Whenever an excited “red'' individual encounters any “green'' individual it changes its color to “green'' in the ground state, 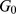. If an excited “red'' individual encounters any other “red'' individual it changes into the “red'' ground state. Consequently, the discrete state space of our model is given by 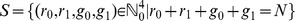, where, naturally, 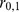 and 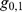 denote the number of “red'' and “green'' robots in the ground and excited state, respectively. The reactions are then 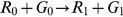
(21)

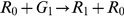
(22)

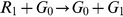
(23)

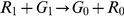
(24)

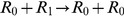
(25)

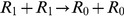
(26)

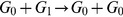
(27)

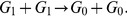
(28)


For clarity of notation, we absorb the reaction rate 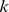, which is given by Eq. (20), into the time unit, i.e. we transform to a new variable 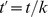 and drop the prime in the notation, and henceforth omit 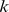 from any equations.

### 1 Non-Markov property of the lumped process

Before we proceed in writing down and solving the chemical Master equation that belongs to Eqs. (21)–(28), we will show that lumped process for the symmetry parameter 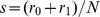 is generally not Markov. The time evolution of 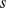 can be obtained by partitioning 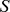 such that the state space of the new process is given by 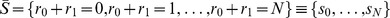. According to Tian *et al.* [34], a continuous-time Markov chain with finite state space 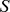 is lumpable, i.e. the lumped process is Markov again, with respect to a particular partition 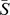 iff 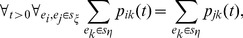
(29)


where 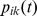 is the transition probability of the original chain to go from state 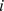 to state 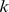 and 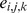 are the members of the state space. Note that, for convenience of notation, we use an arbitrary one-dimensional enumeration of the multi-dimensional state space 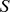.

In words, the lumpability condition Eq. (29) states that the transition probability for any of the states in the original chain that comprise the state 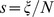 in the lumped chain to go into any state corresponding to 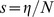 in the lumped chain must be the same, regardless of the source state. We can easily see that this condition is not satisfied. Consider, for example, the situation that half of the robots are initialized as “green'' and have not had the chance to encounter any other robot, i.e. 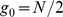 and 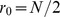, with all other states zero. Clearly, this situation corresponds to 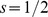 and the transition probability to go into any state with 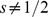 is zero, since no robots are currently in the excited state. However, as the simulation continues, some “red'' robots might encounter “green'' robots and and eventually there might be a number of robots in the state 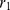. This situation still belongs to the state 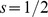 but now the probability to go into a different state 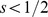 is non-vanishing since these robots might change their color in the next encounter. We conclude that the lumpability is not satisfied and, hence, the lumped process is generally not Markov. It is obvious that this argument extends to the full macroscopic-discrete system Eqs. (11)–(19).

### 2 Elimination of fast variables

We now go about to write down and solve the Master equation for our toy model. The key assumption in our approach is that there is a separation of time-scales between transitions that change the symmetry parameter 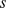 and transitions between the internal states. In other words, for every transition 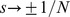, there were many internal transitions between the ground and the excited state. This assumption allows us to eliminate the fast variables that are associated with the internal states and obtain an approximate Master equation for the state parameter, which should be valid on a time scale that is larger than the time scale of internal transitions. Naturally, this assumption is satisfied best if there are many internal states: For each change in 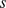 the system needs to undergo a number of internal changes first as robot encounters occur. If the number of internal states is high, we would therefore expect a significant separation of time scales. Our toy model has only one excited state and we expect a model with a larger internal decision depth (e.g. 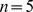 as in the simulations above) will satisfy this key premise reasonably well. Again, we will have to postpone a more comprehensive discussion to further work. Here, we justify our assumption *a posteriori* by validating the results.

The constraint that the total number of robots 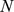 is a conserved quantity allows us to eliminate one of the state variables. We decide to use the state variables 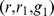 and the obvious relation 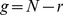, where 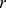 and 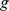 denote the total number of red and green robots, respectively. The state space 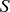 is then 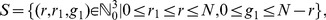
(30)


and the Master equation we strive to solve is 
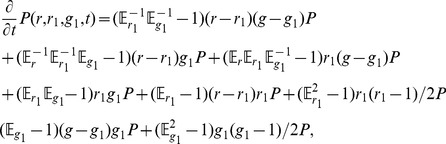
(31)


where 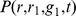 is the probability to find the process in the state 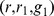 at time 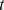. We omit the argument of 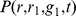 on the right-hand side and define the step operator 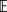 as 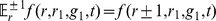 for any function 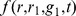 and accordingly for the other state variables 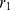 and 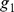 [25].

We now assume that the reactions between the ground and excited states for each species occur much faster than the reactions that change the color of species. This assumption is justified as robots can only change their color, i.e. a change in 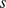 occurs, after they encountered at least 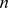 robots of the opposite color. More likely, however, the number of internal changes is much higher, since before a color change occurs, the robots would encounter a number of robots of different colors, each encounter causing a change in internal state. Hence, the higher the decision depth 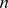 is, the larger the more distinct the separation of time scales will be.

The separation of time scales causes the state 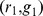, for a given 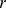, to equilibrate quickly. Following Frankowicz *et al.* [35], we can then eliminate the fast variables 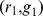. The basic idea is to obtain an equation for the slow variable 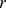 by marginalizing over the fast variables, i.e. 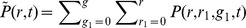. The reduced Master equation for 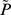 is then 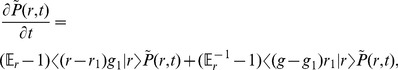
(32)


where we used the abbreviation 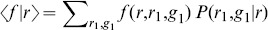 and Bayes' rule 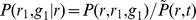.

To close Eq. (32) we now make the assumption that the conditional probability 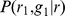 obeys the reduced Master equation [35]

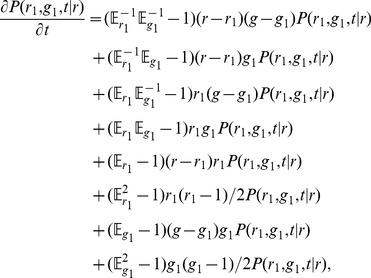
(33)


which follows from Eq. (31) with 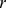 held constant.

We need to solve Equation (33) for the stationary state with 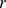 as a parameter. Having obtained the stationary solution 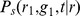, we can compute the conditional averages needed to solve Eq. (32). Since 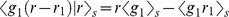 and 
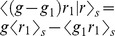
, it is sufficient to compute the moments 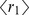, 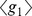, and 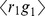. In principle, 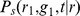 can be obtained directly from Eq. (33) using graph-based methods [36]. However, we avoid this rather cumbersome approach here and perform an 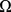-expansion of Eq. (33) instead [25].

### 3 Macroscopic equation for the fast variables

The system-size expansion is an expansion in a parameter 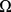, which often stands for the size of the integration domain, and presupposes that, in the macroscopic limit, the probability density is sharply peaked around the macroscopic solution with fluctuations of order 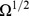. Clearly, this requires that the macroscopic solution is unique and stable such that fluctuations cannot grow. In this subsection, we develop the macroscopic solution and show that it is indeed stable.

The macroscopic equations for the fast variables 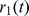 and 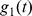 follow immediately from the reaction network Eqs. (21)–(28) under the condition 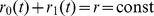. We can easily write down the macroscopic equivalent of Eq. (33) and obtain 
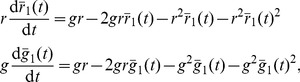
(34)


where we use the scaled variables 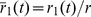 and 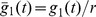.

The initial condition is 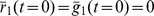 (we assume that there are no excited robots initially) and we can solve Eq. (34), finding 
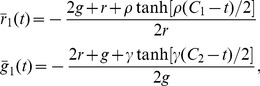
(35)


with 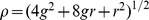, 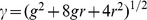, and the integration constants are determined by the initial conditions to be 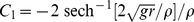 and 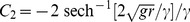.

The stationary solution is unique and can be found in the limit 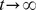 to be 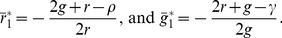
(36)


Linearization of Eq. (34) around the fixed point 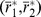 gives the stability matrix 
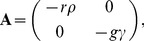
(37)


with the obvious eigenvalues 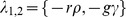. Clearly, the eigenvalues are negative and the fixed point is stable.

### 4 System-size expansion of the fast variables

We solve the Master equation for the fast variables 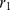 and 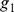 [Eq. (33)] by performing a multi-variate 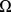-expansion. We assume that the solution exhibits a sharp localized peak and fluctuations around that peak of order 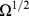, hence 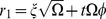, 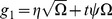, and 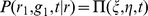. Expanding the step operators and the total time derivative in terms of 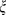 and 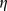 and keeping the terms up to 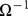 yields the system size expansion [25].

Details of the expansion can be found in Appendix 2. Here we only give the results for the moments of the fast variables: 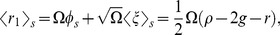
(38)

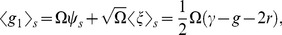
(39)


and 
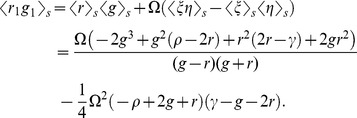
(40)


### 5 The reduced Master equation for the slow variable

We are now in a position to compute the coefficients for the reduced Master equation (32). To allow comparison with the results from the previous section, we first transform Eq. (32) back into a Master equation for the symmetry parameter 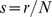, where we also restore the reaction rate 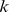: 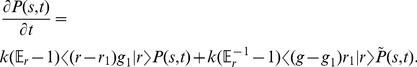
(41)


We need the terms 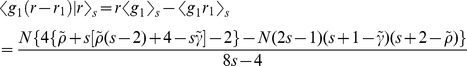
(42)


and 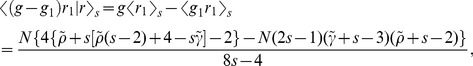
(43)


where we used 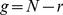, 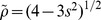, 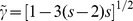 and also set 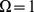.

A comparison with the Master equation (10) and the definition of the stepping functions 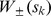 allows us to identify 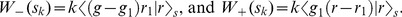
(44)


**Figure 12:**
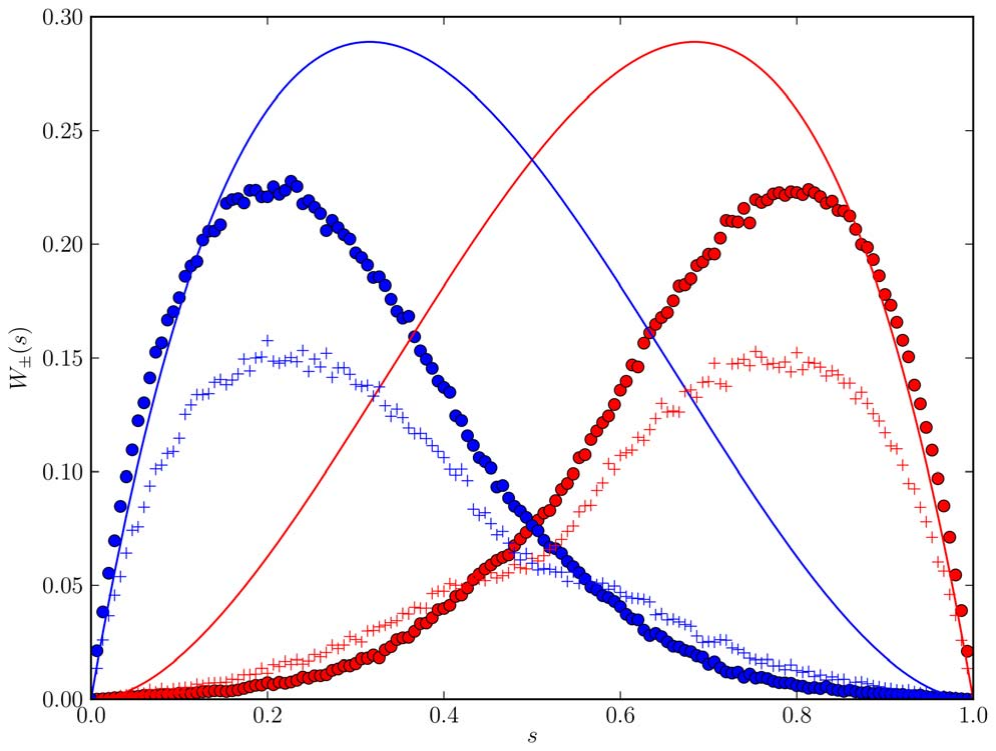
Jump probabilities for the toy model. Jump probabilites 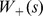 (red curves and markers) and 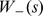 (blue curves and markers) for the toy model [Eqs. (21)–(28)] obtained from the analytic approximation [Eq. (44), curves] and a macroscopic-discrete simulation (circles). For comparison, we include simulation results of the full macroscopic-discrete model (Sec. 2, "plus'' markers).

In Fig. 12, we compare the approximate solution (44) of the Master equation (curve) with a macroscopic-discrete simulation of our toy model (circles) and a macroscopic-discrete simulation of the full model (“plus'' markers). We first observe that the toy model systematically overestimates the tendency to agree on an emerging decision, i.e. 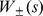 are higher for the toy model. This is easy to explain, as the toy model only allows for two states, the ground state and the excited state, whereas the full model has a decision depth of 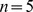. This means that, in the toy model, a robot can be more easily convinced to change its decision. In a similar manner, the approximate solution (curve in the Figure) tends to promote consensus more than the actual macroscopic-discrete system (plus markers). The reason for this is that the time scale for the fast variables to equilibrate, which is given by the eigenvalues of the linear system (Sec. 3) to 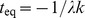. For the macroscopic-discrete toy system (circles), 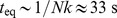 is larger than the typical time scale for a change in the symmetry parameter. The latter can be obtained from 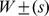 through 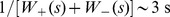 [20]. Hence, the fast variables cannot generally attain equilibrium before the slow variable changes and the fast variable elimination procedure is not fully accurate. We would expect that an analytic solution that allows more than two internal states would cause a more distinct separation of time scales and hence allow better equilibration of the fast states. We speculate that the more detailed model would result in a more accurate approximation of the full Master equation of the toy model and the full macroscopic-discrete simulation. Ultimately, only solving the analytic model for a higher decision depth will provide a definite confirmation.

## Discussion

In this article we have presented a consistent multiscale approach to modelling a typical decision making scenario in swarm robotics. We perform microscopic simulations of the swarm (Section “Microscopic approach'') and conduct physical experiments using a swarm of Kilobots (Section “Kilobot experiments'') to validate our simulations. Following up on previous work [1], we identify a symmetry parameter as the fundamental collective swarm variable and tentatively suggest a continuous-time Markov process to describe its evolution. We derive various macroscopic swarm properties, such as decision time and splitting probability, from the time evolution of the symmetry parameter and compare these results to the data obtained from the simulations and the experiments. Extending upon previous work, we identify approximate spatial homogeneity as a key requirement for this type of modelling and investigate the conditions under which it is valid to assume spatial homogeneity. For this regime we derive a macroscopic-probabilistic model using techniques from chemical kinetics (Section “The macroscopic-discrete approach''). We simulate the macroscopic-discrete model and assess its agreement with the previous results. Finally, we detail an approach to deriving the defining properties of the continuous-time Markov process for the symmetry parameter analytically by solving the macroscopic-probabilistic model (Section “Constructing a time coarse-grained Markov process for the symmetry parameter''). Generally, the decision process is approximated well by the continuous-time Markov process. This is a surprising result given the very restrictive assumptions which are required to obtain the aggregate description.

We investigate the validity of the multiscale approach for different regimes of the microscopic swarm properties. We identify the limiting factors and derive hard quantitative limits for the applicability of the approach. Firstly, depending on the magnitude of the avoidance radius, the assumption of spatial homogeneity breaks down. The mean free path length is the characteristic quantity for spatial homogeneity. If the mean free path length is much smaller than the container dimensions robots will start to form clusters. One case of this is where the avoidance radius is large. Clustering affects the behaviour of the symmetry parameter and, in general, predictions made based on this collective property will be less accurate. Future work will investigate further if spatial structures can be incorporated into a collective description. Secondly, even if spatial homogeneity is satisfied, the lumped process describing the evolution of the symmetry parameter will, in general, not be memoryless and hence not Markov. The symmetry parameter will generally only be well-approximated if the process is ‘almost Markov’, i.e. the memory influences the macroscopic behaviour only marginally. Our analytic toy model suggest that the Markov property will be satisfied better if the decision depth is larger. Future work will extend this analytic model to accomodate a larger decision depth and eventually the full macroscopic-probabilistic model. We believe that this extension, while cumbersome, should not pose any in-principle difficulties.

The analytic treatment of our case study has allowed us to identify the properties under which the proposed empirical parameter estimate of the macroscopic model yields a good approximation of the system's behaviour. For other scenarios these properties would have to be established on a case-by-case basis. How this can be done analytically in other scenarios is beyond the scope of the proposed methodology. In how far the proposed methodology can be generalized thus requires further investigation. Our description hinges on the assumption of spatial homogeneity. Some probabilistic models use homogeneous compartments and incorporate spatial separation by introducing delay times for travelling between compartments [7]. Stochastic delay-differential equations are hard to formulate and even harder to solve and hence we do not expect our approach to work in this case. On the other hand, several studies that assume spatial homogeneity can successfully address problems of collaborative manipulation [8] and task allocation [9]. We believe that our approach can be fruitfully applied to these and related scenarios. Demonstrating the applicability of our approach to these and related scenarios in future work will hopefully allow us to gain increased confidence that this simpler empirical macroscopic method approximates a broad spectrum of application scenarios reliably.

## Appendix

**1 Estimating parameters of the macroscopic process from the simulation output**

**1.1 Probability mass function and SDE parameters.** We can estimate the discrete probability mass function (PMF) from the simulation output  via

(45)

where  denotes the individual simulation,  is an index for the output time in each simulation and the output period  does not change. The index  on  indicates the discrete range of  such that  with . The characteristic, or indicator, function is defined as

(46)

Note that there is no explicit time dependence and the stochastic process is time homogeneous. For any solution  of the FPE [Eq. (2)], the moments of  correspond to the coefficients as [25]

(47)

and

(48)

in the limit . We can use these relations to estimate the drift  and diffusivity  of the SDE by computing

(49)

and

(50)

where, as in Eq. (45),  denotes the experiment and  is an index for the output time. The average is then given by

(51)

and

(52)

The coefficients for the SDE then follow immediately.

**1.2 Aggregate measures.** We can estimate the splitting probability from the simulation results by computing

(53)

i.e. for each bin  we select all simulations that start at  and average the consensus result of these simulations.

We can also compute the splitting probability from Eq. (2) through [27]:

(54)

where the potential  is defined as

(55)

Note that the corresponding Eqs. (5.5.52) and (5.5.53) in [27] incorrectly state that the splitting probability is the integral of  rather than .

The stationary state  can be determined either directly from the simulation output or through the potential :

(56)

where  is a normalization constant. To estimate  from the simulation output, we make use of the ergodicity of the process and compute

(57)

where  is given by Eq. (45) and  is chosen such that most simulations of the ensemble will have attained the stationary solution at .

The time to decision can be extracted directly from the simulation output,

(58)

where the decision time  is the first time where a decision is made, i.e. . We can also calculate  indirectly using the potential  computed in Eq. (55) [27]:

(59)

**1.3 Pair correlation function.** We can estimate the pair correlation function for the “red'' robots from the simulation output using [23]

(60)

where  are the positions of the “red'' individuals and we use a box kernel with bandwidth

(61)

The isotropized set covariance  is designed to minimize boundary effects for large  and is given for a square domain of side length  by [23]

(62)

**1.4 Discrete-state Markov process.** Standard techniques to estimate the aggregate measures of the discrete-state Markov process from the experiment output can be found in text books [20]. We briefly collect the main results here.

The jump probabilities  can be computed using the relation

(63)

between the average time the system stays in state , , and the average number of  steps,  [20]. For each experiment  we then observe the times  (, where  denotes the starting time of the experiment), where a robot changes its color and the corresponding state . We can then extract

(64)

and

(65)

and obtain the jump probabilities using relation (63).

The jump probabilities can be used to compute the splitting probability  and the average decision time  [20]. To this end we employ the recurrence relations

(66)

and

(67)

The splitting probabilities  and  are then given by

(68)

Finally, we have for the average decision time

(69)

**2 System-size expansion**

We perform a sistem-size expansion of the Master equation for the fast variables [Eq. (33)] [25].

Terms of order  need to cancel out which gives the condition

(70)

Evidently, Eq. (70) is satisfied if

(71)

(72)

which are exactly the macroscopic equations Eq. (34) with the stable stationary point  and .

Collecting terms of order  yields a quasi-linear Fokker-Planck equation

(73)

with a Gaussian solution [25]. We can use this equation to obtain expressions for the various moments:

(74)

and

(75)

where we define  and the coefficient matrices are

(76)

and

(77)

We can solve these equations in the stationary state  and finally arrive at the expressions

(78)

(79)

(80)

and

(81)

Transforming back into the original variables we find the results Eqs. (38)–(40).
